# A Comparative Study on the Impact of FIV and FeLV Infection on the Ocular Microbiota in Persian Cats: Insights From Co‐Infection and Single Infections

**DOI:** 10.1155/vmi/8146795

**Published:** 2025-12-19

**Authors:** Ghazal Aftab, Parastou Arab, Pooya Faranoush

**Affiliations:** ^1^ Department of Clinical Sciences, Science and Research Branch, Islamic Azad University, Tehran, Iran, azad.ac.ir; ^2^ Pediatric Growth and Development Research Center, Institute of Endocrinology and Metabolism, Iran University of Medical Sciences, Tehran, Iran, iums.ac.ir

**Keywords:** FeLV, FIV, ocular microbiota

## Abstract

**Background:**

The ocular microbiome of cats infected with feline immunodeficiency virus (FIV) or feline leukemia virus (FeLV) might differ from that of healthy cats. This study aimed to examine and compare the conjunctival bacterial and fungal flora in these groups.

**Methods:**

Bacterial and fungal cultures were conducted from the conjunctiva of 80 Persian cats, categorized into four groups: normal, FIV‐infected, FeLV‐infected, and co‐infected with both FIV and FeLV. PCR assays confirmed the presence of FIV, FeLV, *Chlamydia*, and *Mycoplasma*. The microbiological analysis was compared across the different.

**Results:**

The conjunctival bacterial flora of normal cats was predominantly Gram‐positive, with *Staphylococcus* species as the most common isolates. *Escherichia coli* was absent in the normal group but present in all infected groups, with the highest prevalence in the co‐infected group (17.5%). Co‐infection with FIV and FeLV led to a distinct microbiota with *Streptococcus agalactiae*, *Corynebacterium renale*, *Fusarium*, and *Aspergillus brasiliensis* exclusively found in this group.

**Conclusions:**

The co‐infection of FIV and FeLV significantly alters the conjunctival microbiome, promoting the colonization of specific opportunistic pathogens. These findings may influence the clinical management of cats with these viral infections, especially in combination, and may create a more favorable environment for the growth of certain bacteria and fungi in the conjunctiva.

## 1. Introduction

The body of cats harbors a complex community of microorganisms, collectively referred to as the normal microbiota, which colonizes various anatomical sites and plays a crucial role in host homeostasis. The immune system and microbiota share a bidirectional relationship, wherein immune alterations can lead to microbiota dysbiosis, potentially predisposing the host to opportunistic infections [1–3].

Opportunistic infections predominantly occur in immunocompromised individuals, where disruptions in immune surveillance enable the proliferation of pathogenic microorganisms [4]. In humans, the most well‐characterized immunosuppressive condition is AIDS, resulting from HIV infection. HIV‐induced immune dysregulation has been shown to significantly alter the composition of the fecal, anal, and oral microbiota [5]. Furthermore, HIV‐associated microbiota alterations have been implicated in ocular manifestations, contributing to the pathogenesis of ocular diseases [6].

In felids, feline immunodeficiency virus (FIV) and feline leukemia virus (FeLV) are retroviruses that cause progressive immunosuppression, predisposing infected individuals to opportunistic infections. These viruses primarily affect T‐cell function, leading to immune dysfunction similar to that observed in HIV/AIDS. Immunosuppressed cats exhibit an increased prevalence of fungal and bacterial colonization on their skin and mucosal surfaces, likely due to impaired immune responses [7]. FIV‐induced immunosuppression has been associated with an increased risk of opportunistic infections. Notably, both FeLV and FIV infections have been linked to heightened susceptibility to cryptococcosis and poorer treatment outcomes [8]. Epidemiological data suggest that adult male cats with outdoor access and a history of aggressive interactions are at a greater risk of infection due to behavioral and immunological factors [7].

FeLV infection exhibits three primary clinical outcomes: viral clearance through an effective immune response, persistent viremia, or latent infection. Unlike FIV, which establishes lifelong infection, FeLV can be partially or fully cleared in some individuals, depending on host immune competence [9].

A comprehensive understanding of the ocular microbiota and its alterations in response to FIV and FeLV infection is critical for elucidating the role of these retroviruses in opportunistic ocular infections. This study aims to compare the conjunctival fungal and bacterial flora of FIV‐ and FeLV‐infected cats, including co‐infected individuals, with that of non‐infected counterparts to identify potential microbial shifts associated with viral‐induced immunosuppression.

## 2. Material and Method

This case–control study was conducted on four groups of Persian cats. Age and sex were recorded for all individuals, with an age range of 6–39 months. Each group consisted of 20 Persian cats (40 eyes), totaling 80 cats (160 eyes) across all groups. The cats were referred to the Danesh Veterinary Hospital, which is affiliated with the Islamic Azad University in Tehran, Iran. The study aimed to assess and compare the conjunctival bacterial and fungal flora in cats infected with FIV and FeLV and was conducted between January 2021 and December 2023. All clinical examinations and diagnostic procedures were conducted under the supervision of an internal specialist veterinarian. Written informed consent was obtained from all cat owners before sample collection. ChatGPT (3.5) was used as a language‐assistance tool to improve grammar, spelling, and clarity in the manuscript.

### 2.1. Group Classification and Inclusion Criteria

The first group (Control, C) consisted of healthy cats. Initially, all individuals underwent a comprehensive ophthalmic examination to confirm the absence of ocular diseases. Cats without clinical signs of ophthalmic disorders were further screened for systemic diseases, including feline infectious peritonitis (FIP), feline herpesvirus, feline panleukopenia, endocrine disorders, and internal or external parasitic infections. Blood samples were collected, and initial screening was performed using FIV and FeLV rapid diagnostic kits. Cats testing negative for both retroviruses via rapid tests were further tested using polymerase chain reaction (PCR) to confirm the absence of FIV and FeLV. Only individuals with negative results from both diagnostic methods were included in this group.

The second group (FIV‐positive, FIV) comprised cats infected with FIV. Similar to the control group, these cats were screened for ophthalmic diseases, and any observed abnormalities were documented. They were also assessed for the absence of other viral or bacterial infections. Blood samples were collected, and initial screening was performed using FIV and FeLV rapid tests. If the rapid test was positive for FIV, the sample was further analyzed via PCR for both retroviruses. Only cats confirmed to be FIV‐positive and FeLV‐negative were included in this group.

The third group (FeLV‐positive, FeLV) included FeLV‐infected cats. These cats underwent ophthalmic examinations identical to the previous groups, and any ocular abnormalities were recorded. They were screened for systemic and viral diseases. Blood sampling and rapid testing were conducted, and if FeLV was detected, PCR confirmation was performed for both retroviruses. Only cats testing positive for FeLV and negative for FIV were included in this group.

The fourth group (co‐infected, FIV/FeLV‐positive, +2) consisted of cats infected with both FIV and FeLV. The same screening procedures as in the previous groups were followed. Cats were included in this group only if both the rapid test and PCR confirmed concurrent FIV and FeLV infections.

All examinations, including ophthalmic evaluations, systemic disease screenings, and laboratory diagnostics, were performed under the direct supervision of an internal specialist veterinarian to ensure the accuracy and reliability of the findings.

This structured approach ensured precise group classification while minimizing confounding factors related to other infectious or systemic diseases.

### 2.2. Exclusion Criteria

Cats with ocular diseases unrelated to FIV or FeLV, or those who have received antibiotics within the past 2 weeks, will be excluded. Additionally, cats with systemic illnesses other than FIV or FeLV, previous ocular surgeries or interventions, or those living in uncontrolled environments (e.g., outdoor or multi‐animal households) will not be eligible. Cats with immune‐mediated ocular conditions or known allergies, or those showing severe signs of FIV or FeLV, will also be excluded to minimize confounding factors and ensure a focused study population.

### 2.3. Rapid Test

For all cases, regardless of group classification, the IDEXX SNAP FeLV/FIV Combo rapid test was performed. Blood samples were collected using anticoagulated whole blood or separated serum. The SNAP Combo test detects the p27 antigen for FeLV and antibodies against p24 and gp40 for FIV, providing a rapid and reliable initial screening for retroviral infections.

### 2.4. PCR Detection of FIV and FeLV

Genomic DNA was extracted from blood and conjunctival samples using commercial extraction kits, following the manufacturer’s protocols.

For FIV and FeLV detection, PCR was performed. Specific primers were used under optimized cycling conditions. PCR cycling parameters, electrophoresis, and visualization followed standard protocols (Table 1) [10, 11].

**Table 1:** Demonstrated FIV and FeLV primer sequences of the study.

Organism/virus	Sample type	Target gene	Forward primer (5′ ⟶ 3′)	Reverse primer (5′ ⟶ 3′)	Amplicon size (bp)	PCR conditions
FIV	Blood	gag	5′‐CTG​TGG​AAG​GGT​GAT​GAC​AG‐3′	5′‐ACT​TCT​GCG​CAT​TTC​TTG​CT‐3′	409	95°C 5 min ⟶ 35 cycles: 94°C 30 s, 55°C 30 s, 72°C 45 s ⟶ 72°C 7 min
FeLV	Blood	LTR U3	5′‐AGT​TCT​CAG​TTC​GAG​ACC​AG‐3′	5′‐GAC​CAG​TGA​TCA​AGG​GTG​AG‐3′	202	95°C 3 min ⟶ 35 cycles: 94°C 20 s, 56°C 20 s, 72°C 30 s ⟶ 72°C 5 min

*Note:* Min means minutes.

### 2.5. *Mycoplasma* PCR

*Mycoplasma* detection was performed using a genus‐specific PCR protocol based on the method described by Lauerman et al. [12]. The primers JGMF (5′‐ACA​CCA​TGG​GAG​CTG​GTA​AT‐3′) and JGMR (5′‐CCT​CAT​CGA​CTT​TCA​GAC​CCA​AGG​CAT‐3′), developed by Harasawa et al. [13] in Japan, were used.

The PCR reaction was carried out for 40 cycles, with each cycle consisting of denaturation at 94°C for 30 s, annealing at 55°C for 30 s, and extension at 72°C for 60 s. A final extension step was performed at 72°C for 5 min. This protocol facilitated the detection of *Mycoplasma* in conjunctival samples.

### 2.6. *Chlamydia* Nested PCR

*Chlamydia* detection was conducted using a nested PCR approach targeting variable domains 3 and 4 of the *Chlamydia* omp1 gene, following the method described by Kaltenboeck (1998). The PCR consisted of two sequential reactions: the primary reaction (PRIM3) and the secondary nested reaction (SEC3).

In the PRIM3 reaction, the primers 191CHOMP (5′‐GCITYTITGGGARTGYGGITGYGCIAC‐3′) and CHOMP37 (5′‐TAGAAICKGAATTGIGCRTTIAYGTGIGCIGC‐3′) were used. The PCR reaction included an initial denaturation at 96°C for 10 min, followed by 50 cycles of denaturation at 96°C for 1 s, annealing at 46°C for 1 min, and extension at 72°C for 1 min.

For the SEC3 reaction, the primers 201CHOMP (5′‐GGIGCWGMITTCCAATAYGCICARTC‐3′) and CHOMP336 (5′‐CAAMGTTTCTGGAYTTMAWYTTGTT‐3′) were used. The PCR reaction consisted of 35 cycles, each comprising denaturation at 96°C for 1 s, annealing at 46°C for 1 min, and extension at 72°C for 1 min.

This nested PCR approach allowed for the precise detection of *Chlamydia* in conjunctival samples, providing valuable insights into the presence of infection in the study subjects.

PCR for *Mycoplasma* and *Chlamydia* was performed on conjunctival swabs, whereas FIV/FeLV PCR was conducted on whole‐blood samples.

### 2.7. Electrophoresis and Analysis

PCR products were analyzed by electrophoresis on 1.5% (w/v) agarose gels. Following separation, the gels were stained with ethidium bromide, and DNA bands were visualized using UV transillumination. Standard laboratory precautions were strictly followed to prevent amplicon cross‐contamination throughout the procedure.

### 2.8. Bacterial and Mycologic Culture

Samples for bacterial and fungal cultures were collected using two sterile micro‐swabs. Each swab was transferred to the laboratory and cultured in phosphate‐buffered saline (PBS) [14]. For each eye, two blood agar plates and two MacConkey agar plates were used, with each eye sampled separately. Additionally, one sabouraud dextrose agar (SDA) plate was used for fungal culture.

After 24 h of incubation, bacterial cultures were examined. Isolated cultures were further incubated, and standard biochemical tests were performed for species identification. Cultures were processed within 2 h of collection following the recommended guidelines. Blood agar, MacConkey agar, and thioglycolate broth enrichment medium were used to support the growth of challenging and anaerobic bacteria, while SDA was employed for fungal cultures.

The bacterial cultures were incubated at 35°C–37°C with a 5% CO_2_ atmosphere for 48 h. SDA was incubated at 35°C–37°C for fungal growth. To support the recovery of anaerobic and fastidious bacteria, thioglycolate broth (FTM) was used as an enrichment medium. After inoculation, thioglycolate tubes were immediately sealed with cotton plugs and overlaid with a thin sterile layer of mineral oil to maintain the reduced environment.

The tubes were incubated at 35°C–37°C under anaerobic conditions, created by the inherent reducing activity of sodium thioglycolate and L‐cystine in the medium. The oxidation–reduction indicator (resazurin) was monitored to ensure proper anaerobiosis. Thioglycolate broth cultures were examined daily for turbidity or surface pellicle formation and were incubated for up to 5 days when no initial growth was observed.

After the incubation period, fungal colonies were assessed for colony morphology, and a culture slide test was performed. SDA medium was prepared according to the instructions and divided into culture plates. A centimeter‐sized piece of the culture medium was cut with a sterile scalpel, placed on a sterile glass slide in a glass plate, and inoculated at four points with the grown fungus using a sterile syringe. A sterilized lamella was placed on the inoculated agar, and sterile distilled water was added to prevent drying. The plate was then kept at room temperature for 1 week.

For bacterial colonies, Gram staining was performed, followed by differential diagnostic tests based on the bacterial morphology. The following tests were used to identify specific bacterial species: catalase, OF medium, bacitracin disc, DNase, coagulase, urea hydrolysis, VP, ONPG, and mannitol salt agar.

*Streptococcus* species were identified based on hemolysis patterns and further tested with bacitracin, SXT, and CAMP tests. The Enterobacteriaceae family was identified using TSI, SIM, Simmons citrate, LIA, MR–VP, and urea hydrolysis tests. Nonfermenting G‐bacilli were identified by performing the oxidase test, glucose use in OF medium, and observing pigment production. Gram‐positive bacilli were classified as *Corynebacterium* spp. or diphtheroids based on morphology, spore production, motility, catalase, urea hydrolysis, and CAMP tests. If spores were present, the genus was assigned as *Bacillus*.

Because morphology and biochemical assays have limited discriminatory power for certain taxa, particularly for closely related species, no species‐level identification was assigned when molecular tools or MALDI‐TOF MS were not available. Organisms known to require advanced methods—such as members of the *Staphylococcus intermedius* group (SIG) and species within the *Aspergillus brasiliensis*/*A. niger* complex—were therefore reported at the genus level or as species complexes to avoid over‐specification and ensure taxonomic accuracy.

### 2.9. Data Analysis

To compare each microorganism with the group of cases, a chi‐square statistic test was employed. Significance was determined by *p* values less than 0.05. The data were analyzed using IBM SPSS Version 22.

## 3. Results

**Figure 1:**
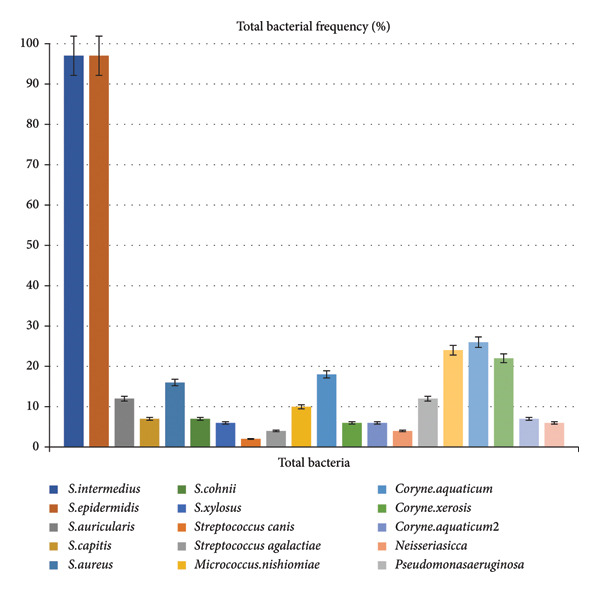
Frequency of bacterial detection from conjunctival samples of Persian cats. The most common bacteria were *Staphylococcus intermedius* and *Staphylococcus epidermidis* (30.3% each), while *Streptococcus canis* was the least detected (0.6%).

A total of 20 Persian cats were studied in each group, with 40 eyes per group, making a total of 80 cats (160 eyes) across all groups. No correlation was found between age or gender and the conjunctival flora. The most common bacteria detected from the conjunctiva of the Persian cats were Gram‐positive bacteria, with *Staphylococcus* species being dominant. *Staphylococcus intermedius* and *Staphylococcus epidermidis* were equally detected in 30.3% of the samples. *Streptococcus canis* was the least detected bacterium, identified in only 0.6% of samples. The frequency of total bacterial detection is shown in Figure 1.

A significant correlation was found between *Staphylococcus aureus* and the co‐infected group, as it was not detected in the normal and FIV groups (0%), but was present in 17.5% of the co‐infected group (*p* value < 0.001). *Streptococcus agalactiae* was another bacterium identified exclusively in the co‐infected group, at a frequency of 1.3% (*p* value < 0.01).

*Corynebacterium renale* was significantly correlated with the co‐infected group, detected in 12.5% of samples. It was also found in other groups, with lower frequencies: 2.5% in the normal group, 5.1% in the FIV group, and 2.5% in the FeLV group. *Neisseria sicca* was identified solely in the FeLV group at a frequency of 5% (*p* value < 0.01).

**Figure 2:**
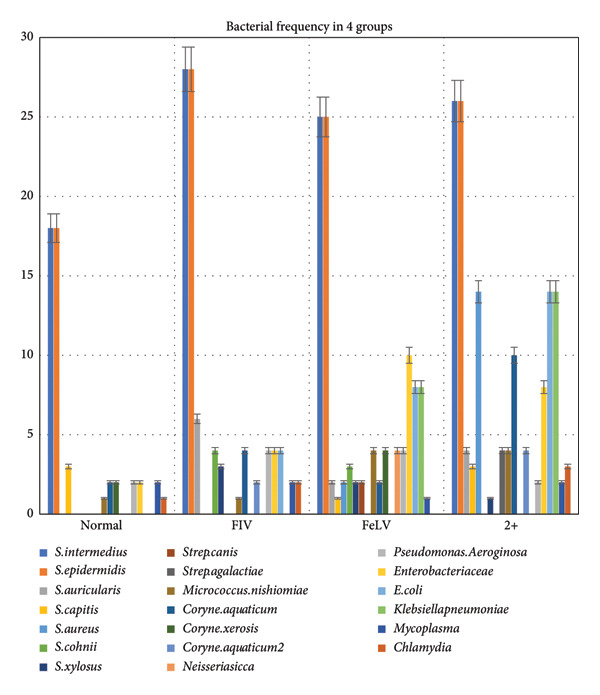
Frequency of bacterial detection across different groups. *Staphylococcus aureus* was significantly associated with the co‐infected group (17.5%) and absent in the normal and FIV groups (*p* < 0.000). *Streptococcus agalactiae* was detected only in the co‐infected group (1.3%, *p* < 0.007). *Corynebacterium renale* was most frequent in the co‐infected group (12.5%) but also found in normal (2.5%), FIV (5.1%), and FeLV (2.5%) groups. *Neisseria sicca* was exclusive to the FeLV group (5%, *p* < 0.007). *E. coli* was absent in the normal group but detected in the FIV (5%), FeLV (8%), and co‐infected (17.5%) groups (*p* < 0.000). *Klebsiella pneumoniae* was found only in the FeLV (10%) and co‐infected (17.5%) groups (*p* < 0.000).

There were no cases of *Escherichia coli* in the normal group, but it was detected in the FIV group (5%), FeLV group (8%), and co‐infected group (17.5%) (*p* value < 0.001). *Klebsiella pneumoniae* was detected only in the FeLV and co‐infected groups, at frequencies of 10% and 17.5%, respectively (*p* value < 0.001). The frequency of bacterial detection in each group is shown in Figure 2.

**Figure 3:**
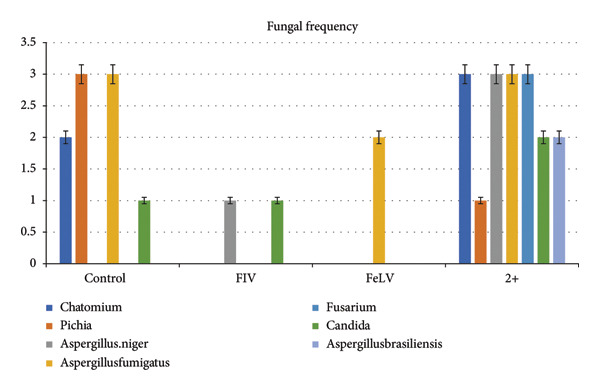
Frequency of fungal species detected in conjunctival samples. *Aspergillus fumigatus* was the most frequently identified fungus, occurring in 2.5% of the samples. *Fusarium* was only present in the co‐infected group (*p*‐value < 0.05). Overall, fungal detection was significantly less frequent than bacterial detection.

The frequency of bacterial detection was notably higher than fungal detection. The most common fungal species identified as *Aspergillus fumigatus* was detected in 2.5% of samples. *Fusarium* was detected exclusively in the co‐infected group (*p* value < 0.05). The frequency of fungal detection is shown in Figure 3.

## 4. Discussion

To the best of our knowledge, this is the first study to simultaneously investigate the conjunctival flora in four groups of Persian cats, categorized based on their FIV and/or FeLV status. Previous studies have primarily focused on the normal ocular flora in healthy domestic shorthair (DSH) and Persian cats. The ocular surface microflora refers to the nonpathogenic, resident bacteria—both aerobic and anaerobic—that naturally inhabit healthy ocular tissues such as the conjunctiva and cornea. These microorganisms likely originate from the skin and colonize the ocular surface, forming a stable ecosystem unless disrupted by disease, trauma, surgery, or antibiotic use [15].

Our study revealed that Gram‐positive bacteria were predominant in the conjunctival bacterial flora of healthy Persian cats, consistent with findings from previous studies on cats and other species. Three studies conducted in Canada, Poland, and Brazil reported *S. epidermidis* as the dominant bacterium in the conjunctival flora of cats [16–18]. Additionally, a study by Aftab et al. on the normal ophthalmic flora of Persian cats over four seasons also found Gram‐positive bacteria to be predominant [15]. Similarly, our study found *Staphylococcus* species, particularly *S. epidermidis* and *Staphylococcus intermedius*, to be the most frequently isolated bacteria across all groups, followed by *Streptococcus* species.

*E. coli* was absent in the normal group but present in all infected groups. The prevalence of *E. coli* in the co‐infected group (cats infected with both FIV and FeLV) was nearly double that observed in the FIV and FeLV groups.

Co‐infection with FIV and FeLV had a more pronounced impact on the conjunctival flora compared to either infection alone. Specifically, *Streptococcus agalactiae*, *C. renale*, *Fusarium*, and *A. brasiliensis* were detected exclusively in the co‐infected group. Additionally, *K. pneumoniae*, found in the FeLV group, showed a significantly higher prevalence in the co‐infected group, nearly double that in the FeLV group.

FIV is a lentivirus transmitted primarily through bites, with feral colonies and roaming tomcats showing the highest infection rates. FIV infection leads to progressive depletion of CD4+ helper T cells, and in advanced stages, a loss of CD8+ cells [19].

FIV can cause ocular disease either directly through viral damage to ocular tissues or indirectly by inducing secondary immune reactions and promoting opportunistic infections. Common ocular manifestations include anterior uveitis, conjunctivitis, lens luxation, and pars planitis [20].

Many cats infected with FIV remain asymptomatic. The ocular diseases associated with FIV are primarily chronic anterior uveitis and conjunctivitis [21].

In one epidemiologic study, 11% of FIV‐positive cats had chronic conjunctivitis [22]. In our study, the FIV group had the highest bacterial detection rate among all groups, with a higher prevalence of both Gram‐positive and Gram‐negative bacteria.

FeLV, an RNA retrovirus, is another significant cause of ocular disease in cats, primarily due to the induction of lymphosarcoma. Other ocular manifestations associated with FeLV include retinal hemorrhages and abnormal papillary motility due to neurological effects [9]. Infection eventually leads to malignant transformation or cytopathic depletion of specific lymphocytic/hematopoietic cell populations. Infection with FeLV can cause infiltrative uveal, conjunctival, orbital, and/or corneal lymphosarcoma. Alternatively, dyscoria or anisocoria can stem from the neurological effects of FeLV on the short ciliary nerves. In addition, FeLV‐induced anemia may lead to secondary retinal hemorrhages [22]. The FeLV group in this study showed a significant correlation with *Neisseria sicca*, which was found in 5% of samples, suggesting a potential association between FeLV infection and this bacterium.

Our study observed a significant correlation between *S. aureus* and the group of Persian cats co‐infected with FIV and FeLV, with a prevalence of 17.5%. This bacterium was absent in both the normal and FIV‐only groups, suggesting that the combined presence of these viruses may predispose the conjunctiva to colonization by *S. aureus*. Similarly, *Streptococcus agalactiae* was exclusively detected in the co‐infected group at a prevalence of 1.3%, further supporting the notion that FIV and FeLV co‐infection creates a favorable environment for specific bacterial species. *C. renale* also showed a higher prevalence in the co‐infected group (12.5%). While it was present at lower frequencies in other groups, this pattern indicates that co‐infection may increase the likelihood of *C. renale* colonization, although it is not exclusive to this group.

These findings align with previous research highlighting the impact of FIV and FeLV co‐infection on the conjunctival microbial isolates. For instance, a study by Marcondes reported that co‐infection with FeLV and FIV was associated with a higher incidence of *Mycoplasma* spp. Infection compared to cats infected with FeLV or FIV alone [23]. Additionally, research by Little observed that cats infected with both FeLV and FIV had increased susceptibility to various secondary infections, including bacterial pathogens [24]. Together with our findings, these studies highlight the complex interactions between viral infections and the conjunctival microbiota in Persian cats.

Our study found that *K. pneumoniae* was present exclusively in the FeLV and co‐infected (+2) groups, with a significantly higher prevalence in the co‐infected group (17.5%) compared to the FeLV group (10%). This observation suggests that FeLV infection, particularly when combined with FIV, may facilitate the colonization of specific bacteria in the conjunctiva.

Regarding fungal flora, our findings indicated a lower frequency of fungal detections compared to bacterial ones. The most commonly identified fungus was *Aspergillus fumigatus*, detected in 2.5% of samples. Notably, *Fusarium* species were exclusively found in the co‐infected group. This exclusive detection in the co‐infected group hints at a possible association between the combined FIV and FeLV infections and the presence of *Fusarium* species.

These results align with existing literature highlighting the impact of FeLV and FIV co‐infection on the conjunctival microbial isolates. For instance, a study comparing the cutaneous and mucosal mycoflora in cats infected with FIV or FeLV to that in noninfected cats found significant differences in fungal populations, suggesting that these viral infections can alter the normal fungal flora of cats [25].

This study provides valuable insights into the conjunctival flora of Persian cats, showing significant correlations between certain bacterial and fungal species and the presence of FIV, FeLV, or both. These findings improve our understanding of the microbial composition of the conjunctiva in Persian cats and have important implications for the management of cats with FIV and FeLV infections. Further research is needed to investigate the mechanisms behind these associations and their clinical significance.

## 5. Conclusion

In conclusion, this study provides valuable insights into the conjunctival flora of Persian cats, particularly in relation to FIV and FeLV infections. Our findings demonstrate that the co‐infection of FIV and FeLV significantly alters the conjunctival microbial isolate composition, with an increased prevalence of specific bacterial species such as *S. aureus*, *Streptococcus agalactiae*, *C. renale*, *K. pneumoniae*, and fungal species like *Fusarium*. These alterations suggest that the combination of FIV and FeLV creates a favorable environment for the colonization of opportunistic pathogens in the conjunctiva. The results also highlight the significant impact of these viral infections on the conjunctival microbial isolates, with implications for the management and treatment of cats affected by FIV and FeLV. Further research is warranted to explore the underlying mechanisms driving these changes and to better understand the clinical consequences of these microbial shifts in immunocompromised cats.

## Ethics Statement

The study was conducted in accordance with ethical guidelines and regulations for animal research. The cats’ welfare and comfort were prioritized throughout the study. Appropriate measures were taken to minimize stress and discomfort during sample collection. The study received approval from the Iran Society for Prevention of Cruelty to Animals, in accordance with the ethical guidelines for laboratory animal studies in Iran. Additionally, the study was conducted in compliance with the ARVO Statement for the Use of Animals in Ophthalmic and Vision Research. Also, all the examinations were conducted with the ethical code of (IR. IAU.SRB.REC.1404.185) certified by the ethical committee of the Islamic Azad University Science and Research Branch. Written informed consent was obtained from all cat owners before sample collection.

## Conflicts of Interest

The authors declare no conflicts of interest.

## Funding

No funding was received for this research.

## Data Availability

The data that support the findings of this study are available from the corresponding author upon reasonable request.
